# TRPV1 and PLC Participate in Histamine H4 Receptor-Induced Itch

**DOI:** 10.1155/2016/1682972

**Published:** 2015-12-24

**Authors:** Tunyu Jian, Niuniu Yang, Yan Yang, Chan Zhu, Xiaolin Yuan, Guang Yu, Changming Wang, Zhongli Wang, Hao Shi, Min Tang, Qian He, Lei Lan, Guanyi Wu, Zongxiang Tang

**Affiliations:** ^1^College of Basic Medicine, Nanjing University of Chinese Medicine, 138 Xianlin Road, Nanjing 210023, China; ^2^College of Life Science, Nanjing Normal University, Nanjing 210046, China; ^3^College of Basic Medicine, Guangxi University of Chinese Medicine, 13 Wuhe Road, Nanning 530200, China

## Abstract

Histamine H4 receptor has been confirmed to play a role in evoking peripheral pruritus. However, the ionic and intracellular signaling mechanism of activation of H4 receptor on the dorsal root ganglion (DRG) neurons is still unknown. By using cell culture and calcium imaging, we studied the underlying mechanism of activation of H4 receptor on the DRG neuron. Immepip dihydrobromide (immepip)—a histamine H4 receptor special agonist under cutaneous injection—obviously induced itch behavior of mice. Immepip-induced scratching behavior could be blocked by TRPV1 antagonist AMG9810 and PLC pathway inhibitor U73122. Application of immepip (8.3–50 *μ*M) could also induce a dose-dependent increase in intracellular Ca^2+^ ([Ca^2+^]_i_) of DRG neurons. We found that 77.8% of the immepip-sensitized DRG neurons respond to the TRPV1 selective agonist capsaicin. U73122 could inhibit immepip-induced Ca^2+^ responses. In addition, immepip-induced [Ca^2+^]_i_ increase could be blocked by ruthenium red, capsazepine, and AMG9810; however it could not be blocked by TRPA1 antagonist HC-030031. These results indicate that TRPV1 but not TRPA1 is the important ion channel to induce the DRG neurons' responses in the downstream signaling pathway of histamine H4 receptor and suggest that TRPV1 may be involved in the mechanism of histamine-induced itch response by H4 receptor activation.

## 1. Introduction

Acute and chronic itch (pruritus) is an unpleasant sensation that elicits the desire or reflex to scratch. It was first defined by Samuel Hafenreffer, a German physician [1]. Pruritus is a common clinical symptom that can accompany skin or systemic diseases, for example, atopic eczema, atopic contact dermatitis, kidney failure, and liver cirrhosis [2]. Pruritogens including amines, proteases, and cytokines have been implicated in the induction of itch such as thymic stromal lymphopoietic protein (TSLP) [3], histamine [4], 5-HT [5], Ser-Leu-Ile-Gly-Arg-Leu (SLIGRL) [6], substance P [7], and IL-31 [8].

Histamine is one of the best-known endogenous mediators for the induction of itch. It is known that histamine receptors, which are found and cloned a class of G protein-coupled receptor so far, can be classified into four referred to as H1–4 [9]. H4 receptor is a new histamine receptor identified in 2000 [10]. Interestingly, some studies have implicated the H4 receptors' involvement in mediating pruritus in mice [11–13]. The intradermally injected H4 receptors agonist 4-methylhistamine could induce itch in mice [12]. In addition, immunohistochemistry and single RT-PCR studies have shown that H4 receptors are expressed in the DRG neurons of humans and rats, and their mRNA have been found in the sensory neuron [14, 15]. Furthermore, the H4 receptor agonist excites the mouse DRG neuron by increasing intracellular free calcium [14]. However, the mechanism of the H4 receptor in DRG neurons—a cluster of nerve cell bodies of peripheral sensory formation including itch—is not yet fully understood. Thus, in the present study, by using cell culture and calcium imaging, we investigated the response properties of DRG neurons by activated histamine H4 receptor downstream signaling pathways and the pruritogens' mechanism of H4 receptor-induction. The results show that the response of DRG by activated H4 receptor was mediated by opening TRPV1 after the stimulated PLC pathway.

## 2. Materials and Methods

### 2.1. Animals and Cultures of Dissociated DRG Neurons

Four-week-old C57BL/6 mice of either sex were deeply anaesthetized with isoflurane and then exsanguinated. The spinal cord was exposed and DRG from all spinal levels were dissected. Isolated ganglia were collected in a cold culture medium containing the following: 90% DMEM-F/12, 10% fetal bovine serum, 100 *μ*g/mL Streptomycin, and 100 U/mL penicillin (Gibco, USA). Ganglia were washed three times with culture medium and enzymatically digested in dispase (5 mg/mL, Gibco, USA) and collagenase type I (1 mg/mL, Gibco, USA) dissolved in Ca^2+^ and Mg^2+^-free Hank's buffered salt solution (HBSS, Gibco, USA) for 30 min at 37°C, as described previously [16, 17]. DRG cells were dissociated by trituration using fire-polished Pasteur pipettes of decreasing tip pore size. Cells were centrifuged at 1200 rpm for 5 min and resuspended in the culture medium, plated on glass cover slips pretreated with 0.5 mg/mL poly-D-lysine (Sigma) and 10 *μ*g/mL laminin (Sigma). Cells were incubated at 37°C in an incubator (95% O_2_ + 5% CO_2_ gas mixture) for 3 h and then flooded with additional culture medium and further incubated at 37°C. Experiments were performed within the next 24 h.

### 2.2. Calcium Imaging

Culture cells on cover slips were loaded with 2 *μ*mol/L fura 2-acetomethoxy ester (Molecular Probes) supplemented with 0.01% Pluronic F-127 (wt/vol) for 40 min in the dark at 37°C. After a three-time wash with HBSS, the cells were imaged by a microscope (Olympus, Japan), and a high-speed continuously scanning monochromatic light source (Polychrome V; Till Photonics, Gräfelfing, Germany) was used for excitation at 340 and 380 nm to detect intracellular free calcium concentration ([Ca^2+^]_i_). Fluorescence intensities at both wavelengths (F340 and F380) were measured every 1–5 s, and images were obtained using PC-based software (C-imaging systems; Hamamatsu Photonic). Test compounds (agonist or antagonist inhibitor) were applied to these cells on the cover glass during scanning. The solution for Ca^2+^ imaging in cultured DRG neurons contained 140 mM NaCl, 5 mM KCl, 1 mM CaCl_2_, 2 mM MgCl_2_, 10 mM Na-HEPES, and 10 mM glucose (pH 7.3). All graphs displaying fura-2 ratios were normalized to the baseline ratio F_340_/F_380_ = (Ratio)/(Ratio_*t*=0_). All data were expressed as means ± SEM. Student's *t*-test was employed for statistical analysis of the data and *P* values of <0.05 were considered significant.

## 3. Results

**Figure 1:**
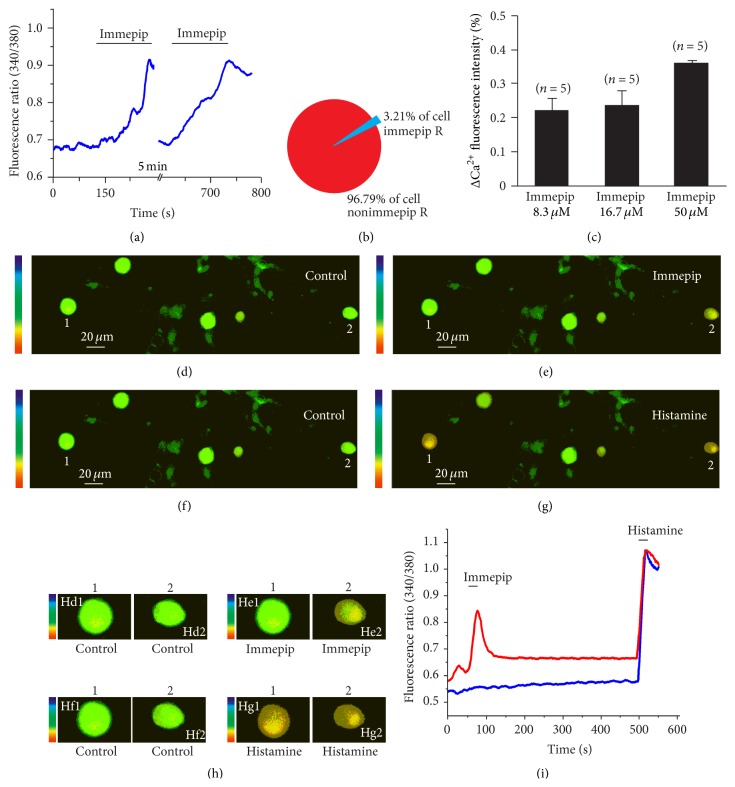
H4 receptor agonist immepip induced an increase in [Ca^2+^]_i_ of the DRG neuron. (a) Representative traces of DRG response to H4 agonist 50 *μ*M immepip twice in 5 min interval. (b) Venn diagram of cell shows the proportion of immepip-response DRG neuron in total test neurons. (c) Histograms show that the DRG neuron exhibited a concentration-dependent increase in [Ca^2+^]_i_ response to 8.3, 16.7, and 50 *μ*M immepip stimulation. (d, e, f, and g) Representative neurons response to 50 *μ*M immepip and 50 *μ*M histamine, (d) control, (e) neuron 2 increase in [Ca^2+^]_i_ by application of immepip, (f) washout, and (g) neurons 1 and 2 increase in [Ca^2+^]_i_ by application histamine. (h) Magnified neuron images of the same neurons in (d–g). Images of Hd1, Hd2 were magnified from neurons 1 and 2 in (d); images of He1, He2 were magnified from neurons 1 and 2 in (e); images of Hf1, Hf2 were magnified from neurons 1 and 2 in (f); images of Hg1, Hg2 were magnified from neurons 1 and 2 in (g). (i) Traces of DRG neurons 1 and 2 from (d–g) response to immepip and histamine; the data show both neuron 1 and neuron 2 respond to histamine (blue line), but only neuron 2 responds to immepip (red line).

**Figure 2:**
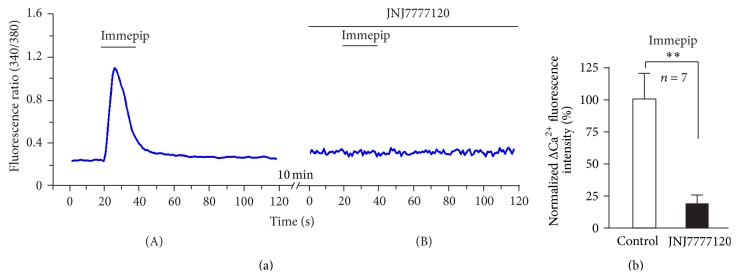
Immepip is a selective agonist of H4 receptor on the DRG neurons. (a) H4 antagonist JNJ7777120 (1 *μ*M) totally blocked the immepip (50 *μ*M)-induced increase in [Ca^2+^]_i_ (*N* = 7). (b) Normalized ΔCa^2+^ fluorescence intensity (%) of the responsive neurons. ^*∗∗*^ *P* < 0.05.

To test the potential role of H4 receptor in the DRG neurons, we first investigated the effects of H4 agonist immepip dihydrobromide (immepip) and histamine to [Ca^2+^]_i_ in dissociated DRG neurons. We applied 50 *μ*M immepip and histamine to the dissociated DRG neurons in a chamber perfusion solution, according to previous studies [14]. Of the 2,305 DRG neuron cultures, 74 (74/2305, 3.21%) showed a remarkable increase of [Ca^2+^]_i_ evoked by H4 agonist immepip; the remaining cells (96.79%) had no response (Figures 1(a) and 1(b)). All these response-neurons to immepip were small-to-medium-sized cells (Figure 1(d)). The number of cells responding did not increase when a high dose of immepip was applied. The immepip-induced increases of [Ca^2+^]_i_ responses on DRG neurons were in a concentration-dependent manner. By using 8.3, 16.6, and 50 *μ*M immepip, the increased [Ca^2+^]_i_ were 0.22 ± 0.03 (*N* = 5), 0.24 ± 0.04 (*N* = 5), and 0.36 ± 0.05 (*N* = 5), respectively (Figure 1(c)). To reveal the relationship between the histamine and H4 agonist immepip on the DRG neurons, in some cases, we perfused the DRG neurons with immepip and histamine. All immepip-activated neurons are the histamine-activated neurons (Figures 1(d)–1(i)). As shown in Figures 1(d)–1(i), immepip only induced Ca^2+^ response on neuron 2, but histamine induced Ca^2+^ response on both neuron 1 and neuron 2 (Figures 1(g), 1(h), and 1(i)). To test whether the immepip is a selective agonist of H4 receptor on the DRG neurons, we investigated the blocked effects of H4 antagonist JNJ7777120 on the immepip-induced response in dissociated DRG neurons. The results show that H4 antagonist JNJ7777120 totally blocked the immepip-induced Ca^2+^ change (*N* = 7) (Figure 2).

**Figure 3:**
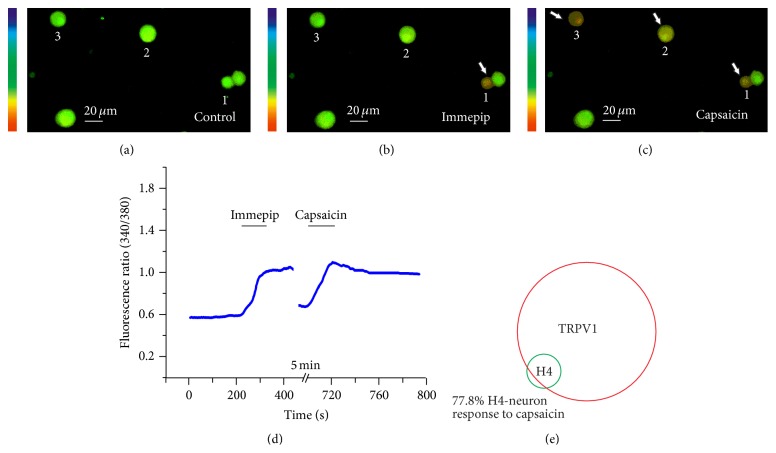
Immepip-sensitive DRG neuron responds to capsaicin. (a) Control, (b) immepip-induced increase in [Ca^2+^]_i_ of DRG neuron 1. (c) Capsaicin-induced increase in [Ca^2+^]_i_ of DRG neurons 1, 2, and 3. (d) Representative traces of another DRG response to immepip (50 *μ*M) and capsaicin (1 *μ*M). (e) Venn diagram showing relative proportion of immepip-sensitive DRG neuron responsive to capsaicin.

It has long been known that most of the neurons' responses to histamine increase in [Ca^2+^]_i_ by application of capsaicin [4]; therefore, to determine more precisely whether immepip-activated neurons also respond to the TRPV1 highly selective agonist capsaicin, we examined the response of cells by the capsaicin-followed immepip. The results indicate that 77.8% (18 of 20) of the neurons that respond to immepip (50 *μ*M) could be activated by application of capsaicin (1 *μ*M) (Figure 3). These results suggest that TRPV1 may be a potential candidate downstream ion channel for activation of H4 receptor on DRG neurons.

**Figure 4:**
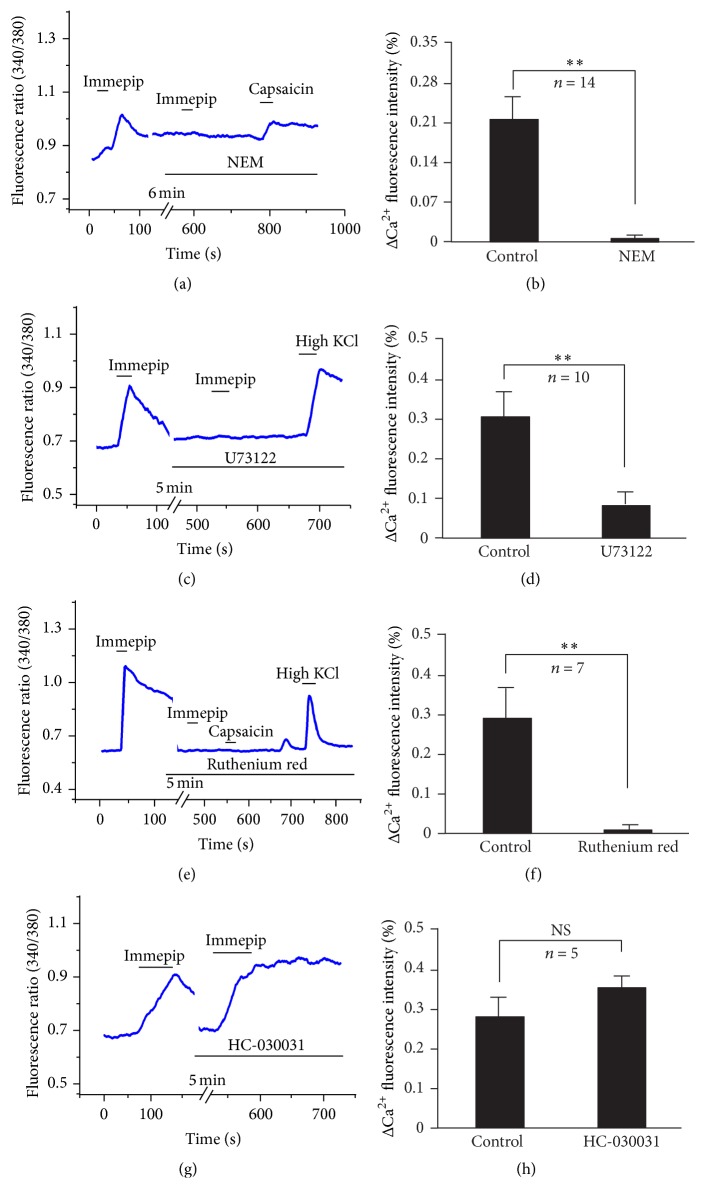
The effects of G protein, PLC, and TRP inhibitor on the immepip-induced increase in [Ca^2+^]_i_ of DRG neurons. (a, c, and e) G protein inhibitor NEM (*N* = 14), PLC inhibitor U73122 (*N* = 10), and TRP channels antagonist ruthenium red (*N* = 7) blocked the immepip-induced increase in [Ca^2+^]_i_, but TRPA1 antagonist HC-030031 (*N* = 7) could not block the immepip-induced increase in [Ca^2+^]_i_ (g). (b, d, f, and h) Mean changes (Δ) *R* (340/380) of the responsive neurons. ^*∗∗*^ *P* < 0.05.

To clarify that G protein is involved in the excitatory effect of H4 agonist immepip on DRG neurons, we tested whether a G protein selective antagonist could block the excitation of immepip-induced response. In 14 detected neurons, N-ethylmaleimide (NEM, 10 *μ*M), a selective G protein blocker [18], blocked the increase in [Ca^2+^]_i_ of immepip-induced response in DRG neurons. [Ca^2+^]_i_ of the DRG neurons increased 21.7% (*N* = 14) by perfusion with immepip. Immepip-induced elevation [Ca^2+^]_i_ of DRG neurons was abolished by preincubated with NEM (Figures 4(a) and 4(b)). In addition, NEM could not block the increase in [Ca^2+^]_i_ of DRG neuron by application of capsaicin (Figure 4(a)). These results indicate that G protein is involved in the H4 agonist immepip-induced excitatory effect.

To determine whether the PLC pathway mediates the neuronal excitation by immepip, a PLC-selective blocker was applied. The results revealed that 10 *μ*M of U73122 could remarkably reduce the increase in [Ca^2+^]_i_ of DRG neurons by application of immepip (0.30 ± 0.05 versus 0.08 ± 0.03, paired *t*-test, *P* < 0.05, and *N* = 10) (Figures 4(c) and 4(d)). These results suggest that immepip induces an increase in [Ca^2+^]_i_ of DRG neurons by stimulating the PLC pathway.

**Figure 5:**
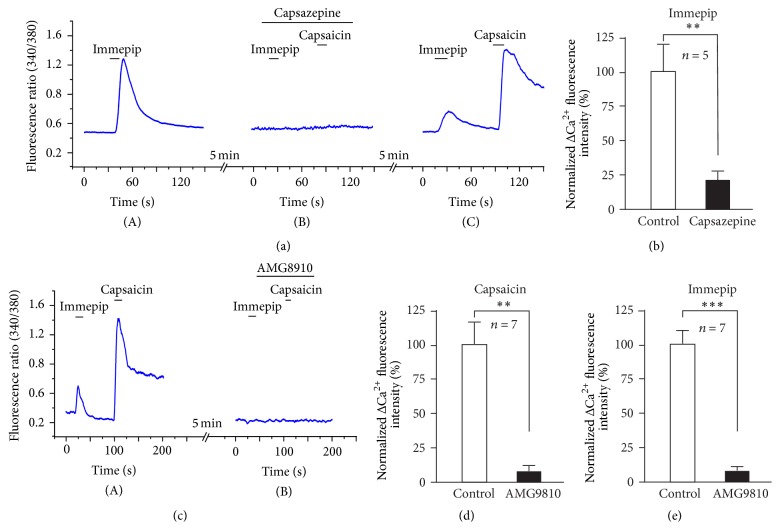
The effects of TRPV1 antagonist capsazepine on the immepip-induced increase in [Ca^2+^]_i_ of DRG neurons. (a) H4 agonist immepip (50 *μ*M) and TRPV1 agonist capsaicin (1 *μ*M)-evoked increase in [Ca^2+^]_i_ were evidently inhibited by TRPV1 antagonist capsazepine (1 *μ*M) (*N* = 5), after washout of capsazepine, the DRG neuron recovery response to immepip and capsaicin. (c) Immepip (50 *μ*M) and TRPV1 agonist capsaicin (1 *μ*M)-evoked increase in [Ca^2+^]_i_ were also obviously blocked by TRPV1 typical antagonist AMG9810 (5 *μ*M) (*N* = 7). (b, d, and e) Normalized ΔCa^2+^ fluorescence intensity (%) of the responsive neurons in the different blockers (^*∗∗*^ *P* < 0.01, ^*∗∗∗*^ *P* < 0.001).

To reveal whether the TRP channel is involved in an excitation action of immepip-induced response on the DRG neurons, we perfused neurons with ACSF containing the TRP channel blocker. As can be seen in Figure 4(e), the results show that a TRP channel antagonist ruthenium red (10 *μ*M), which is known to block TRPV1 and TRPA1, inhibits the increase in [Ca^2+^]_i_ of DRG neurons by an immepip-induced response ([Ca^2+^]_i_ decrease from 0.29 ± 0.08 to 0.076 ± 0.04, Figure 4(f)). In addition, 10 *μ*M of HC-030031, a highly selective TRPA1 antagonist, could not block the immepip-induced calcium influx (0.28 ± 0.05 versus 0.35 ± 0.07, paired *t*-test, *P* = 0.3168, and *N* = 5) (Figures 4(g) and 4(h)). These results indicate that TRPV1 but not TRPA1 is involved in the H4 receptor-mediated effect on the DRG neurons. Furthermore, capsazepine, a highly selective TRPV1 antagonist, could significantly inhibit the immepip-induced excitation on DRG neurons (0.38 ± 0.13 versus 0.09 ± 0.01, paired *t*-test, *P* < 0.05, and *N* = 5) (Figures 5(a) and 5(b)). We also applied a typical TRPV1 antagonist, AMG9810, to test whether the TRPV1 was involved in the immepip-induced response. Immepip- and capsaicin-induced excitation were inhibited by AMG9810 in the same neurons (Figures 5(c), 5(d), and 5(e)).

**Figure 6:**
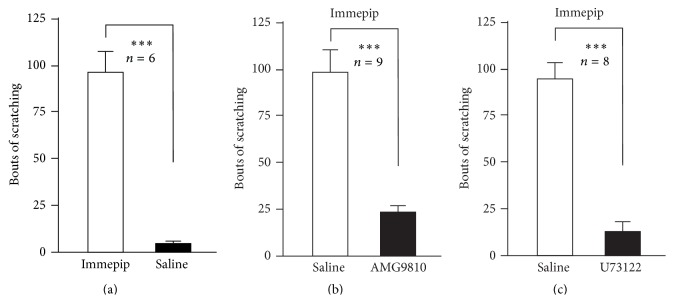
AMG9810 and U73122 inhibited immepip-induced scratching in mice. The histogram shows bouts of scratching by injection of immepip 100 *μ*mol and saline 100 *μ*L/site (*n* = 6) (a). (b) Saline (PBS) 100 *μ*L/site or AMG9810 5 *μ*M 100 *μ*L/site 30 minutes pretreated before immepip subcutaneous injection. Saline (PBS) 100 *μ*L/site or U73122 1 *μ*M 100 *μ*L/site 30 minutes pretreated before immepip subcutaneous injection in (c). The data are presented as mean ± SEM (^*∗∗∗*^ *P* < 0.001).

To confirm the scratching effect of immepip on the histamine H4 receptor-mediated itch, the scratching bouts were counted for 30 minutes after the topical subcutaneous injection of immepip (100 *μ*mol, 100 *μ*L/site) into the nape of the mouse neck. The results show that the immepip induced obvious scratching behavior (96 ± 11 versus 5 ± 1, paired *t*-test, *P* < 0.001, and *N* = 6) (Figure 6(a)). Furthermore, after pretreatment with a typical TRPV1 antagonist, AMG9810, the scratching bouts of the immepip-induced response (98 ± 12, *N* = 9) were significantly blocked (23 ± 3, paired *t*-test, *P* < 0.001, and *N* = 9) (Figure 6(b)). The immepip-induced scratching behavior was also inhibited by U73122. As shown in Figure 6(c), the scratching bouts of immepip decreased from 94 ± 8 to 13 ± 5 (*N* = 8, paired *t*-test, and *P* < 0.001).

## 4. Discussion

Histamine has long been a well-known endogenous pruritogen substance and was richly predominant in peripheral mast cell and basophile granulocytes [2]. H4 receptor is a recently found histamine receptor and is a seven-transmembrane G protein coupled receptor expressed mainly in peripheral bone marrow eosinophils and mast cells [10, 19]. Although there have been extensive studies in recent years about the H4 receptor function in histamine-dependent itch in animal behavior models [11–14], the ionic mechanism and downstream signal pathway of activation of H4 receptor on DRG neuron are still unclear. In the present study, we have revealed that TRPV1 and PLC—but not TRPA1—were involved in the immepip-induced calcium influx on DRG neurons by activation of H4 receptor, demonstrating more cellular details of histamine H4 receptor in sensory neurons.

Phospholipase C (PLC) plays a key role in the signaling pathway links of GPCRs to an intracellular signaling network [20]. In sensory signal transduction, PLC-mediated pathways also play an important role. For example, MrgD, a histamine-independent itch receptor, can be activated by *β*-alanine that couples to an endogenous calcium-activated chloride channel in* Xenopus oocytes* by the PLC pathway [21, 22]. The PLC signaling is also required for TSLP-induced itch in epithelial cell-derived atopic dermatitis [3]. PLC*β*3, a PLC isoform, is required to mediate the itch sensation in response to histamine acting on the histamine H1 receptor in C-fiber nociceptive neurons [23]. Similarly, in the present study, PLC antagonist U73122 totally blocked the immepip-induced excitation of the DRG neurons by activation of the histamine H4 receptor (Figure 4). These results suggest that the PLC pathway is involved in the histamine H4 receptor intracellular signaling of DRG neurons.

TRPV1 is a nonselective cation channel present predominantly in primary small sensory neurons and is activated by the pungency of capsaicin and acid or at temperatures over 43°C [24]. TRPV1 can also be indirectly activated by several other substances through activation on their specific receptors and by initiating various intracellular signaling pathways, such as histamine [4, 25], leukotriene B4 [26], or IL-31. TRPV1 has also been shown to be involved in histamine-induced scratching by activation of the histamine H1 receptor that can be activated by a phospholipase A2 pathway [4, 25]. But the interaction between TRPV1 and the histamine H4 receptor is still not understood. In this study, the highly selective TRPV1 antagonist capsazepine blocked the response of DRG neurons upon activation of the histamine H4 receptor. Furthermore, the TRP channel blocker, ruthenium red, can also block the response by activation of the histamine H4 receptor. The increase in [Ca^2+^]_i_ of DRG neurons by immepip-induced response could not be inhibited by the highly selective TRPA1 antagonist HC03003. These results indicate that TRPV1 is a functional downstream ionic channel of intracellular signaling by activation of the DRG histamine H4 receptor.

What is more, TRPV1 can be coupled to the PLC intracellular signaling pathway. First, TRPV1 currents were sensitized and activated by bradykinin, which is an inflammatory mediator, and by extracellular cations [27, 28]. Furthermore, TRPV1 can be activated by the byproduct of PLC activation, such as diacylglycerol, one of two classical second messengers of PLC [29]. TRPV1 can also be modulated by the substrates of PLC, which include the membrane phospholipid PIP2, inositol 1,4,5-trisphosphate (IP3), and diacylglycerol. They could dually modulate the sensitization and desensitization of the TRPV1 channel [30]. In this study, PLC and TRPV1 may be jointly involved in the excitation of DRG neurons by the activation of histamine H4 receptor. More studies need to be conducted to ascertain how the downstream byproduct of PLC activates TRPV1.

In summary, by activation of the histamine H4 receptor on mouse DRG neurons, immepip induced an increase in [Ca^2+^]_i_ via the intracellular PLC signaling pathway and TRPV1 but not TRPA1. By activation of the histamine H4 receptor, histamine may directly modulate the sensation from endogenous and exogenous mediators [31–33]. Considering that histamine is a well-known and definite itch causing substance [34–36], the histamine H4 receptor may constitute a critical part of histamine-induced itch in peripherals. It is presumed that the function of the histamine H4 receptor on the DRG neurons will help us clearly understand the mechanisms of histamine-dependent itch.

## References

[1] Ikoma A., Steinhoff M., Ständer S., Yosipovitch G., Schmelz M. (2006). The neurobiology of itch. *Nature Reviews Neuroscience*.

[2] Bautista D. M., Wilson S. R., Hoon M. A. (2014). Why we scratch an itch: the molecules, cells and circuits of itch. *Nature Neuroscience*.

[3] Wilson S. R., Thé L., Batia L. M. (2013). The epithelial cell-derived atopic dermatitis cytokine TSLP activates neurons to induce itch. *Cell*.

[4] Shim W.-S., Tak M.-H., Lee M.-H. (2007). TRPV1 mediates histamine-induced itching via the activation of phospholipase A2 and 12-lipoxygenase. *The Journal of Neuroscience*.

[5] Nojima H., Carstens E. (2003). 5-hydroxytryptamine (5-HT)2 receptor involvement in acute 5-HT-evoked scratching but not in allergic pruritus induced by dinitrofluorobenzene in rats. *The Journal of Pharmacology and Experimental Therapeutics*.

[6] Shimada S. G., Shimada K. A., Collins J. G. (2006). Scratching behavior in mice induced by the proteinase-activated receptor-2 agonist, SLIGRL-NH2. *European Journal of Pharmacology*.

[7] Andoh T., Nagasawa T., Satoh M., Kuraishi Y. (1998). Substance P induction of itch-associated response mediated by cutaneous NK1 tachykinin receptors in mice. *The Journal of Pharmacology and Experimental Therapeutics*.

[8] Cevikbas F., Wang X., Akiyama T. (2014). A sensory neuron-expressed IL-31 receptor mediates T helper cell-dependent itch: involvement of TRPV1 and TRPA1. *The Journal of Allergy and Clinical Immunology*.

[9] Shim W.-S., Oh U. (2008). Histamine-induced itch and its relationship with pain. *Molecular Pain*.

[10] Leurs R., Chazot P. L., Shenton F. C., Lim H. D., De Esch I. J. P. (2009). Molecular and biochemical pharmacology of the histamine H4 receptor. *British Journal of Pharmacology*.

[11] Bell J. K., McQueen D. S., Rees J. L. (2004). Involvement of histamine H_4_ and H_1_ receptors in scratching induced by histamine receptor agonists in BalbC mice. *British Journal of Pharmacology*.

[12] Dunford P. J., Williams K. N., Desai P. J., Karlsson L., McQueen D., Thurmond R. L. (2007). Histamine H_4_ receptor antagonists are superior to traditional antihistamines in the attenuation of experimental pruritus. *The Journal of Allergy and Clinical Immunology*.

[13] Cowden J. M., Zhang M., Dunford P. J., Thurmond R. L. (2010). The histamine H4 receptor mediates inflammation and pruritus in Th2-dependent dermal inflammation. *Journal of Investigative Dermatology*.

[14] Rossbach K., Nassenstein C., Gschwandtner M. (2011). Histamine H 1, H 3 and H 4 receptors are involved in pruritus. *Neuroscience*.

[15] Strakhova M. I., Nikkel A. L., Manelli A. M. (2009). Localization of histamine H_4_ receptors in the central nervous system of human and rat. *Brain Research*.

[16] Kim A. Y., Tang Z., Liu Q. (2008). Pirt, a phosphoinositide-binding protein, functions as a regulatory subunit of TRPV1. *Cell*.

[17] Liu Q., Tang Z., Surdenikova L. (2009). Sensory neuron-specific GPCR Mrgprs are itch receptors mediating chloroquine-induced pruritus. *Cell*.

[18] Shapiro M. S., Wollmuth L. P., Hille B. (1994). Modulation of Ca^2+^ channels by PTX-sensitive G-proteins is blocked by N-ethylmaleimide in rat sympathetic neurons. *The Journal of Neuroscience*.

[19] Nakaya M., Takeuchi N., Kondo K. (2004). Immunohistochemical localization of histamine receptor subtypes in human inferior turbinates. *The Annals of Otology, Rhinology, and Laryngology*.

[20] Marinissen M. J., Gutkind J. S. (2001). G-protein-coupled receptors and signaling networks: emerging paradigms. *Trends in Pharmacological Sciences*.

[21] Liu Q., Sikand P., Ma C. (2012). Mechanisms of itch evoked by beta-alanine. *The Journal of Neuroscience*.

[22] Zhuo R.-G., Ma X.-Y., Zhou P.-L. (2014). Mas-related G protein-coupled receptor D is coupled to endogenous calcium-activated chloride channel in *Xenopus oocytes*. *Journal of Physiology and Biochemistry*.

[23] Han S.-K., Mancino V., Simon M. I. (2006). Phospholipase C*β* 3 mediates the scratching response activated by the histamine H1 receptor on C-fiber nociceptive neurons. *Neuron*.

[24] Caterina M. J., Schumacher M. A., Tominaga M., Rosen T. A., Levine J. D., Julius D. (1997). The capsaicin receptor: a heat-activated ion channel in the pain pathway. *Nature*.

[25] Kim B. M., Lee S. H., Shim W. S., Oh U. (2004). Histamine-induced Ca^2+^ influx via the PLA_2_/lipoxygenase/TRPV1 pathway in rat sensory neurons. *Neuroscience Letters*.

[26] Fernandes E. S., Vong C. T., Quek S. (2013). Superoxide generation and leukocyte accumulation: key elements in the mediation of leukotriene B_4_-induced itch by transient receptor potential ankyrin 1 and transient receptor potential vanilloid 1. *The FASEB Journal*.

[27] Premkumar L. S., Ahern G. P. (2000). Induction of vanilloid receptor channel activity by protein kinase C. *Nature*.

[28] Ahern G. P., Brooks I. M., Miyares R. L., Wang X.-B. (2005). Extracellular cations sensitize and gate capsaicin receptor TRPV1 modulating pain signaling. *The Journal of Neuroscience*.

[29] Woo D. H., Jung S. J., Zhu M. H. (2008). Direct activation of transient receptor potential vanilloid 1(TRPV1) by diacylglycerol (DAG). *Molecular Pain*.

[30] Rohacs T., Thyagarajan B., Lukacs V. (2008). Phospholipase C mediated modulation of TRPV1 channels. *Molecular Neurobiology*.

[31] Bíró T., Tóth B. I., Marincsák R., Dobrosi N., Géczy T., Paus R. (2007). TRP channels as novel players in the pathogenesis and therapy of itch. *Biochimica et Biophysica Acta: Molecular Basis of Disease*.

[32] Liu T., Ji R.-R. (2013). New insights into the mechanisms of itch: are pain and itch controlled by distinct mechanisms?. *Pflügers Archiv*.

[33] Simons F. E. R., Simons K. J. (2011). Histamine and H_1_-antihistamines: celebrating a century of progress. *The Journal of Allergy and Clinical Immunology*.

[34] Akiyama T., Carstens E. (2013). Neural processing of itch. *Neuroscience*.

[35] Davidson S., Giesler G. J. (2010). The multiple pathways for itch and their interactions with pain. *Trends in Neurosciences*.

[36] Kremer A. E., Feramisco J., Reeh P. W., Beuers U., Oude Elferink R. P. J. (2014). Receptors, cells and circuits involved in pruritus of systemic disorders. *Biochimica et Biophysica Acta*.

